# High Kynurenine:Tryptophan Ratio Is Associated With Liver Fibrosis in HIV-Monoinfected and HIV/Hepatitis C Virus–Coinfected Women

**DOI:** 10.1093/ofid/ofz281

**Published:** 2019-06-11

**Authors:** Ani Kardashian, Yifei Ma, Michael T Yin, Rebecca Scherzer, Olivia Nolan, Francesca Aweeka, Phyllis C Tien, Jennifer C Price

**Affiliations:** 1 Department of Medicine, University of California Los Angeles, Los Angeles, California; 2 Department of Medicine, University of California San Francisco, San Francisco, California; 3 Department of Medicine, College of Physicians and Surgeons, Columbia University, New York, New York; 4 School of Medicine, College of Physicians and Surgeons, Columbia University, New York, New York; 5 Medical Service, Department of Veteran Affairs Medical Center, San Francisco, California, USA

**Keywords:** HIV/HCV coinfection, kyn/trp ratio, liver fibrosis

## Abstract

**Background:**

Tryptophan catabolism, measured by the kynurenine:tryptophan (kyn/trp) ratio, is associated with gut microbiota alterations in people with HIV (PWH). We examined the association of the kyn/trp ratio with liver fibrosis in women with/without HIV infection.

**Methods:**

The plasma kyn/trp ratio was measured in 137 HIV-monoinfected, HIV/hepatitis C virus (HCV)–coinfected, and uninfected women in the Women’s Interagency HIV Study. Fibrosis was estimated using FIB-4 in all participants and vibration-controlled transient elastography liver stiffness measurement (LSM) in a subset (n = 83). We used multivariable linear regression to evaluate the associations of infection status and kyn/trp ratio with relative differences in fibrosis estimates.

**Results:**

The median kyn/trp ratio (interquartile range) was 0.056 (0.045–0.066) in HIV/HCV-coinfected, 0.038 (0.032–0.046) in HIV-monoinfected, and 0.031 (0.025–0.034) in uninfected women (*P* < .001). After adjustment for sociodemographic, lifestyle, and metabolic factors, HIV monoinfection and HIV/HCV coinfection were associated with 37% (95% confidence interval [CI], 9% to 73%) and 164% (95% CI, 100% to 250%) greater FIB-4, respectively. When kyn/trp ratio was included, higher kyn/trp ratio was associated with greater FIB-4 (27% per kyn/trp doubling; 95% CI, 5% to 53%), and the associations of HIV monoinfection (29% per kyn/trp doubling; 95% CI, 2% to 63%) and HIV/HCV coinfection (123% per kyn/trp doubling; 95% CI, 63% to 203%) with greater FIB-4 were attenuated. Among those with LSM, higher kyn/trp ratio was associated with greater LSM (43% per kyn/trp doubling; 95% CI, 15% to 79%) in multivariable analysis.

**Conclusions:**

The kyn/trp ratio is elevated in PWH and is associated with greater liver fibrosis. Tryptophan catabolism may modify the relationships between HIV, HCV, and fibrosis.

Chronic liver disease has emerged as a leading cause of morbidity and mortality in people with HIV (PWH) [1]. Indeed, in the era of antiretroviral therapy (ART), liver-related deaths occur at a rate 10 times higher than that of the general population [2]. PWH who are coinfected with hepatitis C virus (HCV) experience accelerated liver fibrosis progression and develop hepatocellular carcinoma (HCC) at younger ages compared with those with HCV alone [3, 4].

Alterations in the gut microbiome have also been identified as an important determinant in liver disease pathogenesis in PWH. HIV depletes CD4+ lymphocytes in the gut, promoting increased microbial permeability or “leakiness” in the gastrointestinal mucosa and allowing bacterial translocation of lipopolysaccharide (LPS) into the portal and systemic circulation [5–7]. Elevated levels of circulating LPS lead to an increased state of immune activation and subsequent inflammation. Circulating LPS, along with LPS binding protein and CD14, bind to Toll-like receptor 4 and transcription factors on Kupffer cells in the liver, upregulating proinflammatory and profibrogenic cytokines [8]. Additionally, independent of LPS, elevated levels of the fungal polysaccharide beta-D-glucan due to gut fungal colonization have been linked to greater systemic immune activation in PWH [9].

Tryptophan catabolism is involved in a variety of immune responses and has been proposed as an important pathway contributing to HIV pathogenesis. In particular, indoleamine 2,3-dioxygenase (IDO), an intracellular enzyme expressed in the gut and numerous other tissues that catabolizes tryptophan to kynurenine, has been implicated in promoting systemic inflammation and increased microbial translocation in HIV-monoinfected and HIV/HCV-coinfected persons [10–12]. Tryptophan 2,3-dixoygenase (TDO), which is primarily expressed in the liver and brain, also catabolizes tryptophan; the relative contributions of IDO and TDO to increased tryptophan catabolism in the setting of HIV and HCV are unclear [13]. In HIV, high IDO activity and tryptophan catabolism lead to depletion of CD4+ gut lymphocytes, promoting microbial and fungal translocation (which may also contribute to the induction of IDO activity) and persistent immune dysregulation [9, 11, 12]. The plasma kynurenine:tryptophan (kyn/trp) ratio reflects activation of this pathway and has been linked to neurocognitive dysfunction and depression, cardiovascular disease, and increased mortality in HIV infection [12, 14–16]. Furthermore, the kyn/trp ratio was recently established as a predictor of HIV reservoir size, suggesting that this pathway plays a key role in HIV pathogenesis [17].

Tryptophan catabolism has also been shown to play a role in HCV infection [18]. Persons with chronic HCV infection have higher IDO levels than healthy controls, and clearance of HCV has been shown to normalize IDO levels [19–21]. Greater IDO levels have also been identified in HCV-moninfected persons with cirrhosis and HCC as compared with healthy volunteers or HCV-monoinfected persons without cirrhosis [19]. However, the potential role of IDO level in mediating liver fibrosis in PWH has not been examined. Understanding the complex relationship of HIV, HCV, microbial translocation, and liver fibrosis may help to prioritize interventions directed at improving liver disease among PWH. Therefore, we aimed to determine the association of IDO activity, as measured by the plasma kyn/trp ratio, with liver fibrosis in PWH with or without HCV coinfection, compared with uninfected controls.

## METHODS

### Study Participants

The Women’s Interagency HIV Study (WIHS) is a multicenter prospective cohort study that enrolled a total of 4982 women (3678 HIV-infected and 1304 HIV-uninfected) between 1994 and 2015 from 11 US cities. Baseline sociodemographic characteristics and HIV risk factors were similar between HIV-infected and uninfected women [19,20]. An institutional review board approved the study protocols and consent forms, and each study participant gave written informed consent. Every 6 months, participants completed a comprehensive physical examination, provided biological specimens, and completed an interviewer-administered questionnaire.

From December 2003 through July 2015, 152 women at the San Francisco WIHS site underwent magnetic resonance (MR) spectroscopy to investigate the contributions of HIV, HCV, and metabolic factors to hepatic steatosis and fibrosis. HIV infection was determined by positive HIV antibodies, and HCV infection was determined using the HCV antibody test, followed by confirmatory HCV RNA testing. Participants who had negative HIV and HCV antibody levels were considered to be uninfected. Testing for HIV and HCV was done centrally using a standardized definition to assess infection status. Patients with evidence of hepatitis B surface antigenemia, prior HCV treatment, or history of decompensated cirrhosis were excluded from enrollment. Of these women, 10 were excluded from our analysis because plasma samples were not available for kyn/trp ratio testing. Only 5 women were HCV-monoinfected; they were excluded due to the small sample size, leaving a total of 137 women included in the analysis (59 HIV-monoinfected, 42 HIV/HCV-coinfected, and 36 HIV-uninfected).

### Ascertainment of Body Composition and Metabolic Factors

Visceral adipose tissue (VAT) and abdominal subcutaneous adipose tissue (SAT) were calculated as the mean volume (cm^3^) of the visceral compartments by magnetic resonance imaging (MRI) of the L2/3, L3/4, and L4/5 intervertebral disk levels. Body mass index (BMI) was calculated from height and weight in kilograms per meter squared (kg/m^2^). We estimated insulin resistance using the Homeostasis Model Assessment (HOMA-IR; fasting insulin [microU/mL] × glucose [mg/dL]/405). Hepatic steatosis was assessed by magnetic resonance spectroscopy (MRS) and quantified as liver fat fraction (LFF), as previously described [22].

### Measurement of Kynurenine: Tryptophan Ratios

Plasma tryptophan and kynurenine levels were measured using plasma samples stored at –70ºC from all participants within 6 months of their MR spectroscopy. Liquid chromatography–tandem mass spectrometry was used to assess kynurenine and tryptophan levels, as previously described [23]. Kynurenine and tryptophan were measured at the same visit as the fibrosis-4 score (FIB-4) and were both expressed in nanomoles per milliliter.

### Fibrosis Measurements

Liver fibrosis was estimated using FIB-4 as our surrogate for fibrosis because it has been validated as a noninvasive surrogate for fibrosis in patients with HIV/HCV coinfection, including in the WIHS [24, 25]. FIB-4 is calculated using the following formula: (age in years × AST [U/L])/(platelets × 10^9^/L × (ALT [U/L])^1/2^). It has a specificity of 97% and positive predictive value of 65% in HIV/HCV-coinfected persons at detecting advanced fibrosis [24]. FIB-4 was estimated using data collected during the visit closest to and within 6 months of MR spectroscopy. FIB-4 scores were considered invalid if the AST or ALT was >10 times the upper limit of normal or if platelet counts were <25 × 10^9^ cells/L, as these extreme values are unlikely to be due to chronic liver fibrosis and more likely to be caused by acute hepatitis or another disease process. Probable cirrhosis was defined as FIB-4 >3.25 or AST to platelet ratio index (APRI) >2 [26]. In 83 of the 137 women, liver stiffness measurement (LSM) was performed using FibroScan (Echosens, Paris, France) by trained operators while patients were in a fasting state. LSM was performed at a median (interquartile range [IQR]) of 57.5 (10.5–176) days from the date of kyn/trp ratio measurement. Measurements were considered successful if they were acquired without abnormal vibration shape or propagation. Liver stiffness was expressed in kiloPascals (kPa) as the median value of successful measurements. Only scans with at least 10 successful measurements and an interquartile range of <30% were considered reliable.

### Covariates

Our primary predictors were kyn/trp ratio, HIV, and HCV infection status. Additional covariates included sociodemographic factors (age, race/ethnicity), lifestyle factors (history of injection drug use, alcohol use [none, >0–7 drinks/week, 7–12 drinks/week, >12 drinks/week]); current marijuana use, current smoking, and metabolic factors (waist circumference, body mass index, MRI-measured VAT and abdominal SAT volume, MRS-measured steatosis, and HOMA-IR). HIV-related factors included current CD4, nadir CD4, current HIV RNA, history of clinical AIDS, and current use of ART.

### Statistical Analysis

We compared sociodemographic and clinical characteristics among women stratified by HIV and HCV status using the *t* test or Kruskal-Wallis test for continuous variables and the chi-square test or Fisher exact test for categorical variables. To better understand factors associated with the kyn/trp ratio, we also compared participant characteristics stratified by kyn/trp ratio tertile.

To determine whether HIV monoinfection and HIV/HCV coinfection were independently associated with fibrosis, we used a multivariable linear regression model, controlling for patients’ sociodemographic characteristics, lifestyle factors, and metabolic parameters. We also constructed models restricted to participants with HIV infection to assess the association of HIV-related factors with fibrosis. We next added kyn/trp ratio to the models to determine the independent association of kyn/trp ratio with fibrosis and to evaluate whether adjustment for kyn/trp ratio altered the associations of HIV, HCV, and fibrosis. The final covariates in the entire cohort multivariable model were age, race, alcohol use, marijuana use, IDU, steatosis, VAT, HIV monoinfection, and HIV/HCV coinfection +/- kyn/trp ratio. The final covariates in the model restricted to participants with HIV infection were the same as the entire cohort model, plus CD4 count and HIV viral load. A secondary correlation analysis was performed to assess whether there was an association between kyn/trp ratio and fibrosis, as measured by FIB-4, by infection status. We next performed an interaction analysis to determine whether HIV or HIV/HCV modified the association with kyn/trp ratio and fibrosis (or vice versa). Finally, we repeated the analysis in the subgroup of 83 women with available LSM.

Fibrosis measures were right-skewed and log-transformed to normalize their distributions. The effects (coefficients) were therefore presented as the percentage difference per unit change in the corresponding covariates. In the models with missing covariates, multiple imputation with the Markov Chain Monte Carlo method was used to impute missing covariates, with 25 repetitions [27, 28]. The same set of sociodemographic, lifestyle, and metabolic characteristics, as well as HIV/HCV infection status, was used to create imputation models. Multiple imputation estimates of model parameters were computed by averaging the estimates from all the repetitions of the imputation algorithm, and the variance and confidence interval of these estimates were computed using Rubin’s combining formula [29]. All analyses were performed using the SAS system, version 9.4 (SAS Institute Inc., Cary, NC).

## RESULTS

### Cohort Characteristics

Overall, the median age of the cohort (IQR) was 50 (42–56) years, 55% were African American, and the median BMI (IQR) was 28 (23–33) kg/m^2^. The sociodemographic, metabolic, and clinical characteristics of 137 participants stratified by HIV and HCV status are shown in Table 1. HIV/HCV-coinfected women (n = 42) and HIV-monoinfected women (n = 59) were older than uninfected women (n = 36). HIV/HCV-coinfected women were more likely to be current smokers and to have ever used injection drugs than HIV-monoinfected or uninfected women. HIV-uninfected women had greater BMI and waist circumference than HIV-monoinfected or HIV/HCV-coinfected women. As expected, HIV/HCV-coinfected women had higher AST and ALT levels and lower platelet counts. Compared with HIV-monoinfected women, HIV/HCV-coinfected women had lower median CD4 counts (current and nadir) and higher median HIV viral loads and were more likely to have a detectable HIV viral load. One HIV/HCV-coinfected participant had a history of HCC.

**Table 1.** Demographic and Clinical Characteristics of Women by Infection Status^a^

Median (IQR) or No. (%)	HIV-Monoinfected (n = 59)	HIV/HCV-Coinfected (n = 42)	HIV-Uninfected (n = 36)	*P* Value
Demographics				
Age, y	50 (46–55)	52 (47–57)	42 (37–53)	.002
Race				.269
AA	28 (48)	26 (62)	21 (58)	
White	14 (24)	6 (14)	7 (19)	
Hispanic	8 (14)	9 (21)	5 (14)	
Other	9 (15)	1 (2)	3 (8)	
Lifestyle				
Alcohol				.982
None	26 (44)	21 (53)	19 (53)	
0–7 drinks/wk	26 (44)	15 (38)	13 (36)	
7–12 drinks/wk	4 (7)	2 (5)	2 (6)	
>12 drinks/wk	3 (5)	2 (5)	2 (6)	
Current smoker	19 (32)	28 (67)	15 (42)	.003
Current marijuana user	22 (38)	10 (25)	18 (49)	.098
IDU ever	5 (9)	27 (64)	5 (14)	<.001
Metabolic				
BMI, kg/m^2^	27 (23–33)	24 (22–32)	32 (29–38)	.001
Waist circumference, cm	95 (85–102)	88 (82–102)	104 (93–118)	.002
Steatosis (LFF)	0.01 (0.01–0.04)	0.02 (0.01–0.04)	0.02 (0.01–0.1)	.080
VAT, cm^3^	145 (104–187)	120 (85–197)	169 (99–212)	.503
Abdominal SAT, cm^3^	296 (210–429)	255 (191–348)	440 (201–523)	.067
HOMA-IR	1.87 (1.21–3.62)	2.89 (1.78–4.41)	2.22 (1.13–3.45)	.292
Liver-related				
AST level, U/L	19 (16–26)	41 (28–65)	20 (15–22)	<.001
ALT level, U/L	15 (13–22)	30 (21–52)	16 (13–22)	<.001
Platelet count	250 (213–290)	192 (158–254)	278 (230–359)	<.001
FIB-4	0.98 (0.79–1.53)	2.17 (1.24–3.38)	0.63 (0.57–0.92)	<.001
Presence of cirrhosis	2 (3.4)	11 (26.2)	0 (0)	<.001
HIV-related factors				
HIV RNA, copies/mL	48 (20–139)	80 (41–871)	N/A	.031
HIV RNA undetectable	35 (59)	12 (29)	N/A	.002
CD4, /μL	567 (445–752)	406 (216–638)	N/A	.005
CD4 nadir	229 (100–288)	148 (108–236)	N/A	<.001
% currently on ART	49 (83)	33 (79)	N/A	.57
History of clinical AIDS	21 (36)	26 (62)	N/A	.01

Abbreviations: AA, African American; APRI, AST to platelet ratio index; ALT, alanine aminotransferase; ART, antiretroviral therapy; AST, aspartate aminotransferase; FIB-4, fibrosis-4 score; HCV, hepatitis C virus; HOMA-IR, Homeostatic Model Assessment of Insulin Resistance; IDU, injection drug use; IQR, interquartile range; LFF, liver fat fraction; RNA, ribonucleic acid; SAT, subcutaneous adipose tissue; U/L, units per liter; VAT, visceral adipose tissue.

^a^A *t* test or Kruskal-Wallis test was used to compare characteristics for continuous variables, and the chi-square test or Fisher exact test was used for categorical variables. Missing values: waist circumference: 1 HIV/HCV-coinfected; steatosis: 7 HIV-monoinfected, 10 HIV-uninfected; VAT and abdominal SAT: 9 HIV-monoinfected, 12 HIV/HCV-coinfected, 10 HIV-uninfected; HOMA-IR: 22 HIV-monoinfected, 16 HIV/HCV-coinfected, 9 HIV-uninfected; HIV viral load: 2 HIV-monoinfected; CD4 count: 1 HIV-monoinfected, 1 HIV/HCV-coinfected, 28 HIV-uninfected.

### Factors Associated With Kyn/Trp Ratio

We compared sociodemographic, metabolic, and clinical characteristics of study participants, stratified by tertile of kyn/trp ratio (Supplementary Table 1). Persons with a higher kyn/trp ratio were older on average and were more often smokers and injection drug users, relative to those with a lower kyn/trp ratio. In addition, those with a higher kyn/trp ratio had higher levels of AST and ALT and lower platelet counts. In HIV-infected women, those with a higher kyn/trp ratio had lower current CD4 counts and lower CD4 nadirs.

**Figure 1.**
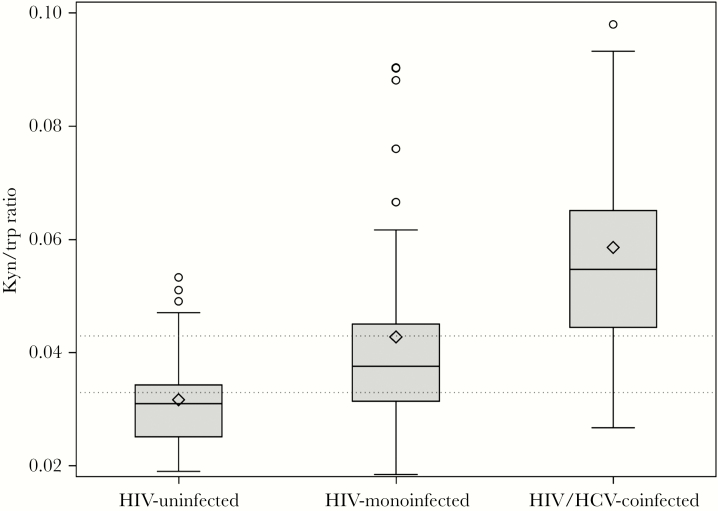
Median kyn/trp ratio by infection status. Normal range of kyn/trp ratio is <0.027 µmole/µmole [30]. Abbreviation: HCV, hepatitis C virus.

A comparison of kyn/trp ratio levels by infection status found that levels were highest in HIV/HCV-coinfected women (median [IQR], 0.056 [0.045–0.066]), followed by HIV-monoinfected women (median [IQR], 0.038 [0.032–0.046]), and lowest in uninfected women (median [IQR], 0.031 [0.025–0.034]; *P* < .001) (Figure 1).

### Associations of HIV, HIV/HCV, and Kyn/Trp Ratio With FIB-4

HIV/HCV-coinfected and HIV-monoinfected women had higher FIB-4 scores than uninfected women, with median FIB-4 scores (IQR) of 2.17 (1.24–3.38) and 0.98 (0.79–1.53), respectively, vs 0.63 (0.57–0.92; *P* < .001). On multivariable analysis, HIV monoinfection (37% greater FIB-4; 95% confidence interval [CI], 9% to 73%) and HIV/HCV coinfection (164% greater FIB-4; 95% CI, 100% to 250%) were associated with greater FIB-4 (Table 2), as was older age (30% greater FIB-4 per 10 years; 95% CI, 16% to 45%). There was no association of VAT (–4% per doubling; 95% CI, –16% to 10%) with FIB-4 and little association of MRS-measured steatosis (2% per doubling; 95% CI, –5% to 9%) and injection drug use (15%; 95% CI, –11% to 48%) with FIB-4. When the kyn/trp ratio was added to the model, a higher kyn/trp ratio was associated with greater FIB-4 (29% per kyn/trp doubling; 95% CI, 2% to 63%), and the associations of HIV monoinfection and HIV/HCV coinfection were attenuated but remained statistically significant: 29% (95% CI, 2% to 63%) and 123% (95% CI, 63% to 203%), respectively.

**Table 2.** The Associations of HIV Monoinfection and HIV/HCV Coinfection With FIB-4 in the Total Cohort (n = 137) in Multivariable Models Adjusted for Demographic, Lifestyle, Metabolic Factors, and Kyn/Trp Ratio

	Adjusted Model^a^		Adjusted Model With Kyn/Trp Ratio^b^	
Variables	% Effect (95% CI)	*P* Value	% Effect (95% CI)	*P* Value
HIV monoinfection	37 (9 to 73)	.008	29 (2 to 63)	.035
HIV/HCV coinfection	164 (100 to 250)	<.001	123 (63 to 203)	<.001
Kyn/trp ratio	N/A	N/A	27 (5 to 53)	.013

Abbreviations: CI, confidence interval; FIB-4, fibrosis-4 score; HCV, hepatitis C virus; IDU, injection drug use; kyn/trp ratio, kynurenine:tryptophan ratio; VAT, visceral adipose tissue.

^a^Adjusted for age, race, alcohol, marijuana, IDU, steatosis, and VAT, in addition to the factors listed above.

^b^Adjusted for kyn/trp ratio, plus all factors in previous model.

We used the kyn/trp ratio as an indicator of tryptophan catabolism for our analyses, consistent with prior data reporting kyn/trp as a valid marker of tryptophan catabolism. However, to evaluate whether changes in kynurenine or tryptophan levels had a greater impact on FIB-4, we also evaluated each covariate independently in multivariable models. A greater kynurenine level (per doubling) was associated with 29.6% greater FIB-4 (95% CI, 8.3% to 55%). However, a greater tryptophan level (per doubling) was associated with only a 6.9% greater FIB-4 and was not statistically significant (95% CI, –18.8% to 40.7%).

**Figure 2.**
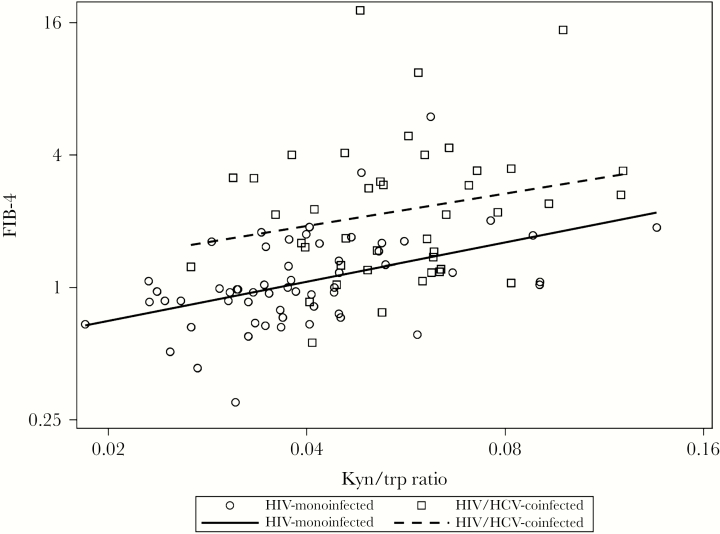
Correlation of kyn/trp ratio with fibrosis-4 score (FIB-4) levels by infection status. No significant association was observed between kyn/trp ratio and FIB-4 in uninfected persons. Abbreviation: HCV, hepatitis C virus.

We next evaluated correlations of the kyn/trp ratio with levels of FIB-4, stratified by infection status (Figure 2). Among HIV-monoinfected women, a higher kyn/trp ratio was associated with greater FIB-4 (Spearman *r *= .51; *P *< .001). A higher kyn/trp ratio also appeared to be associated with greater FIB-4 in HIV/HCV-coinfected women, but the association did not reach statistical significance (*r* = .17; *P* = .17). In contrast, the kyn/trp ratio did not appear to be associated with greater FIB-4 in uninfected women (*r* = –.11; *P* = .51). Because we found differences in the correlation of the kyn/trp ratio and FIB-4 by infection status, we next constructed models examining the interaction effects of HIV and HCV infection status and kyn/trp ratio while controlling for other covariates. The result showed that each doubling of kyn/trp ratio was associated with a 38% (95% CI, 8% to 77%; *P* = .009) higher FIB-4 in HIV-monoinfected women and a 33% (95% CI, –3% to 81%; *P* = .08) higher FIB-4 in HIV/HCV-coinfected women, but a 20% lower FIB-4 (95% CI, –49% to 28%; *P* = .36) in uninfected women. However, the overall interaction effect did not reach statistical significance (*P* = .07).

In multivariable analysis limited to HIV-infected women, HCV coinfection (68%; 95% CI, 26% to 124%) and older age (43%; 95% CI, 22% to 68%) were associated with greater FIB-4 (Table 3). When kyn/trp ratio was added to the model, a higher kyn/trp ratio was again associated with greater FIB-4 (29% greater FIB-4 per kyn/trp ratio doubling; 95% CI, 4% to 59%), and the association of HCV coinfection was attenuated but remained statistically significant (52%; 95% CI, 14% to 104%). Higher CD4 counts were associated with lower FIB-4 (–5.6% per 100 copies; 95% CI, –9.8% to –1.1%), but the effect was no longer statistically significant after adding kyn/trp ratio to the model (–4.5%; 95% CI, –8.8% to 0%).

**Table 3.** Factors Associated With FIB-4 in HIV-Infected Persons (n = 101), With and Without Adjustment for Kyn/Trp Ratio

	Adjusted Model^a^		Adjusted Model With Kyn/Trp Ratio^b^	
Variables	% Effect (95% CI)	*P* Value	% Effect (95% CI)	*P* Value
Age (per 10 y)	43 (22 to 68)	<.001	37 (17 to 61)	<.001
HIV/HCV coinfection	68 (26 to 124)	<.001	52 (14 to 104)	.005
CD4 count	–5.6 (–9.8 to –1.1)	.016	–4.5 (–8.8 to 0)	.050
Kyn/trp ratio	N/A	N/A	29 (4 to 59)	.020

Abbreviations: CI, confidence interval; FIB-4, fibrosis-4 score; HCV, hepatitis C virus; IDU, injection drug use; kyn/trp ratio, kynurenine:tryptophan ratio; VAT, visceral adipose tissue.

^a^Adjusted for age, race, alcohol, marijuana, IDU, steatosis, VAT, and HIV viral load, in addition to the factors listed above.

^b^Adjusted for kyn/trp ratio, plus all factors in the previous model.

### Association of HIV, HIV/HCV, and Kyn/Trp Ratio With Liver Stiffness Measurement

In our multivariable analysis using LSM to measure fibrosis among the subset of women with LSM available (39 HIV-monoinfected, 20 HIV/HCV-coinfected, 24 HIV-uninfected), a higher kyn/trp ratio was significantly associated with increased LSM (43% greater LSM per kyn/trp doubling; 95% CI, 15% to 79%) (Supplementary Table 2). Similar to our models with FIB-4 as the outcome, HIV/HCV coinfection was associated with a 94% higher LSM (95% CI, 40% to 170%), and after additional adjustment for kyn/trp ratio, the association of HCV coinfection was attenuated but remained significant (47%; 95% CI, 3% to 110%). However, neither age nor HIV monoinfection was associated with greater LSM. In our analysis limited to HIV-infected women, HCV coinfection (51% greater LSM per kyn/trp doubling; 95% CI, 8% to 111%) and kyn/trp ratio (52% greater LSM per kyn/trp doubling; 95% CI, 18% to 95%) remained associated with higher LSM.

## DISCUSSION

In this study of women with or at risk for HIV infection, we found that the kyn/trp ratio was higher in HIV-infected women and that a higher kyn/trp ratio was independently associated with greater liver fibrosis, as measured by both FIB-4 and LSM. Consistent with other studies, we found that HIV infection, and particularly HIV/HCV coinfection, was associated with greater fibrosis, even after adjustment for demographic, lifestyle, adipose tissue, and metabolic factors. The associations of HIV monoinfection and HIV/HCV coinfection with liver fibrosis were attenuated when kyn/trp ratio was included in the multivariable models, suggesting that kyn/trp ratio may modify the association of HIV infection and increased fibrosis.

Our findings of increased fibrosis in HIV-infected women, particularly those with HIV/HCV coinfection, are consistent with prior data demonstrating more rapid fibrosis progression in the setting of HIV infection [31–33]. Several potential pathways have been implicated in the pathogenesis of liver fibrosis in HIV. First, HIV is associated with systemic inflammation, which may result in increased cytokine production and cellular infiltration within hepatocytes, contributing to liver inflammation and fibrosis [34]. Second, HIV itself has been shown to play a direct role in liver fibrosis progression. Hepatic stellate cells express key HIV co-receptors, including CCR5 and CXCR4, that allow HIV entry into hepatic stellate cells, with subsequent activation and secretion of proinflammatory cytokines [35, 36]. Third, alterations of the gut barrier and subsequent microbial translocation in HIV infection have been implicated as promoting liver fibrosis [34, 37, 38]. HIV-associated CD4+ lymphocyte depletion can lead to gut mucosal “leakiness,” allowing bacterial translocation of LPS and other microbial products, which reach the liver via the portal vein. Increased levels of LPS have been shown to activate hepatic stellate cells, leading to chronic immune activation and liver fibrosis [37, 38].

The role of altered tryptophan catabolism in promoting microbial translocation in HIV infection and contributing to HIV pathogenesis has received increasing attention recently [10–12, 14]. In an evaluation of treated and untreated PWH, a dysbiotic mucosally adherent microbial community, as measured by analysis of rectosigmoid biopsies, was strongly associated with both a high kyn/trp ratio and high IL-6 [10]. Moreover, the authors demonstrated that several bacteria within this dysbiotic microbial community had the capacity to metabolize tryptophan through the kynurenine pathway. These tryptophan catabolites have been shown to inhibit the differentiation of IL-17-secreting CD4+ T cells, promoting gut “leakiness” and activating a cycle of immune dysregulation in HIV [39, 40]. Finally, elevated beta-D-glucan associated with intestinal fungal colonization also correlates with increasing kyn/trp ratio and immune activation markers in PWH [9]. Dysregulation in the T-regulatory immune system from microbial translocation through increased tryptophan catabolism has been linked to increased cardiovascular disease, depression, and mortality in PWH [12, 14–16].

Although the IDO enzyme, which is widespread and expressed in the gut and numerous other tissues, is primarily responsible for tryptophan catabolism, tryptophan can also be catabolized by another enzyme, TDO. TDO is mainly expressed in the liver of mammals [41]. Its primary role is in the degradation of tryptophan at excess levels and in controlling plasma tryptophan homeostasis through preventing toxic accumulation in the plasma and tissues [41]. Because the liver is involved in clearance of plasma tryptophan during inflammatory states and TDO is expressed in the liver, TDO may play a role in mediating liver inflammation and subsequent fibrosis [13]. However, the contribution of TDO to fibrosis can only be assessed through measurements of TDO protein expression in liver biopsies, which were not available for our cohort.

To our knowledge, our study is the first to show an association between high kyn/trp ratio and greater liver fibrosis in PWH, with or without HCV infection. This association was observed using 2 separate surrogates for fibrosis, FIB-4 and LSM, and persisted even after controlling for demographic, lifestyle, metabolic factors, and infection status. One prior study by Jenabian et al. similarly found a correlation of kynurenine levels with fibrosis, as measured by APRI, in unadjusted analysis among HIV/HCV-coinfected individuals with high APRI scores, but it did not examine whether there was a correlation in the HIV-monoinfected and healthy control groups. There also were several differences from our study: persons with middle-range fibrosis (APRI scores, 0.5 to 1.5) were excluded from the study and only those on long-term ART with suppressed HIV viral loads were included [42]. Interestingly, we found that the associations of HIV monoinfection and HIV/HCV coinfection with fibrosis were attenuated after adding kyn/trp ratio into our multivariable model. Moreover, kyn/trp ratio and fibrosis were correlated in our HIV-infected groups, but not in our HIV-uninfected group. This suggests a potential role of the kyn/trp ratio as a mediator of liver fibrosis in PWH.

We also observed that HIV/HCV-coinfected women had higher kyn/trp ratio levels as compared with HIV-monoinfected women. HCV infection itself may play a role in microbial translocation in PWH, which then may contribute to accelerated fibrosis progression. In a retrospective evaluation of HCV-monoinfected and uninfected persons, IDO levels were significantly higher in the group with chronic HCV as compared with healthy controls, and they were greatest in those with HCV-associated cirrhosis and HCC [19]. Among PWH, HCV coinfection is associated with significant elevations in the monocyte activation markers soluble CD14 and IL-6, suggesting that HCV independently contributes to intestinal barrier damage [43]. In addition, HCV eradication in HIV/HCV-coinfected individuals results in significant decreases in microbial translocation markers, including LPS [42, 44].

It is also possible that a reciprocal relationship exists; fibrosis due to HCV may lead to greater immune activation and tryptophan catabolism. In an analysis of 120 HIV-monoinfected, HCV-monoinfected, and HIV/HCV-coinfected women, liver fibrosis was an important mediator of elevations in soluble CD14 in the HCV-infected groups [45]. In the previously mentioned cross-sectional study by Jenabian et al., a significant correlation was found between HIV/HCV coinfection and kynurenine levels only in individuals with a high APRI, but not in those with a low APRI [42]. The study authors concluded that fibrosis itself may be the primary driver of altered tryptophan catabolism in HIV/HCV coinfection. Given the cross-sectional design of our study, we could not determine the causality of the association between kyn/trp ratio and fibrosis. Further prospective studies are therefore needed to better understand this association. They also noted in a subgroup of HIV/HCV-coinfected persons who were treated with direct-acting antiviral therapy and achieved HCV sustained virologic response (SVR) that APRI scores improved 6 months after treatment but kyn/trp ratios did not. Their findings suggest that despite improvement in liver fibrosis markers, the systemic immune activation and inflammatory liver damage from tryptophan dysregulation may persist even after SVR. We excluded patients who had achieved HCV treatment; therefore, we could not assess the impact of HCV cure in our cohort. Furthermore, whether kyn/trp ratio eventually normalizes at a longer post-SVR follow-up time is unknown and requires further investigation.

There were several other limitations to our study. We used FIB4, a noninvasive serum surrogate, to assess fibrosis, which has been well-validated in HIV/HCV-coinfected persons, but less so in the absence of HCV or other known liver disease [24, 26]. However, we used LSM, which is a more accurate assessment of liver fibrosis, in a sizable subset of the cohort and similarly found that a higher kyn/trp ratio was associated with greater liver stiffness, supporting our findings in the larger cohort. In contrast, when we used LSM as the measure of fibrosis, HIV monoinfection was no longer associated with greater fibrosis. This may be due to the smaller sample size of women with LSM and the lack of sufficient power to detect an association. Another possibility is that LSM is a more accurate measure of fibrosis than FIB-4, which incorporates AST and ALT values and therefore may also reflect liver inflammation regardless of fibrosis. Another potential limitation, as in all observational studies, is the possibility of residual or unmeasured confounders for which we were unable to control. For example, although we adjusted for many factors in our analysis, there are likely differences by HIV and HCV status in other unmeasured parameters. Additionally, given that there were only patients in our cohort who had a history of HCC, we could not evaluate the potential association between IDO activity and liver cancer. Finally, our study was conducted in a cohort of women, and therefore our findings may not be generalizable to men with HIV. The study’s strengths include extensive characterization of metabolic and HIV-related risk factors and the inclusion of HIV- and HCV-uninfected controls.

In conclusion, we found that kyn/trp ratio levels were higher in HIV-infected women and were associated with greater fibrosis. The elevated kyn/trp ratio in the setting of HIV may, in part, explain the association of HIV and HIV/HCV infections with increased fibrosis. These findings are among the first to provide evidence that tryptophan catabolism through the kynurenine pathway may be clinically relevant in liver fibrosis progression in the setting of HIV. Whether this pathway is causally associated with fibrosis will need to be elucidated in future prospective studies, but our data highlight the kyn/trp ratio as a potential noninvasive biomarker and therapeutic target in liver fibrosis among PWH.

## Supplementary Data

Supplementary materials are available at *Open Forum Infectious Diseases* online. Consisting of data provided by the authors to benefit the reader, the posted materials are not copyedited and are the sole responsibility of the authors, so questions or comments should be addressed to the corresponding author.

ofz281_suppl_supplementary_table_1

ofz281_suppl_supplementary_table_2
