# Supplementary material for: High Kynurenine:Tryptophan Ratio Is Associated With Liver Fibrosis in HIV-Monoinfected and HIV/Hepatitis C Virus–Coinfected Women
Source: Open Forum Infect Dis. 2019 Jun 11;6(7):ofz281. doi: 10.1093/ofid/ofz281 (PMC6612851; doi:10.1093/ofid/ofz281)
Supplement: ofz281_suppl_supplementary_table_1 [file ofz281_suppl_supplementary_table_1.docx]

Supplementary table 1. Demographic and clinical characteristics of women by KTR tertiles*

| **Median (IQR) or n (%)** | **KTR tertile 1** | **KTR tertile 2** | **KTR tertile 3** | **P-value** |
| --- | --- | --- | --- | --- |
| KTR | 0.0293  (0.0248-0.0318) | 0.0402  (0.0366-0.0426) | 0.0629  (0.0531-0.0775) | <0.001 |
| **Demographics** |  |  |  |  |
| Age | 43 (39-52) | 50 (43-55) | 55 (50-58) | <0.001 |
| Race |  |  |  | 0.502 |
| AA | 29 (64%) | 22 (47%) | 24 (53%) |  |
| White | 7 (16%) | 12 (26%) | 8 (18%) |  |
| Hispanic | 5 (11%) | 7 (15%) | 10 (22%) |  |
| Other | 4 (9%) | 6 (13%) | 3 (7%) |  |
| **Lifestyle** |  |  |  |  |
| Alcohol |  |  |  | 0.579 |
| None | 21 (47%) | 21 (46%) | 24 (55%) |  |
| 0-7 drinks/week | 19 (42%) | 17 (37%) | 18 (41%) |  |
| 7-12 drinks/week | 2 (4%) | 5 (11%) | 1 (2%) |  |
| >12 drinks/week | 3 (7%) | 3 (7%) | 1 (2%) |  |
| Current smoker | 18 (40%) | 17 (36%) | 27 (60%) | 0.049 |
| Current marijuana user | 18 (40%) | 16 (35%) | 27 (60%) | 0.87 |
| IDU ever | 4 (9%) | 16 (35%) | 16 (36%) | 0.001 |
| **Metabolic** |  |  |  |  |
| BMI (kg/m2) | 29 (25-33) | 28 (24-35) | 25 (22-31) | 0.113 |
| Waist Circumference (cm) | 99 (88- 107) | 95 (86-104) | 89 (82-105) | 0.214 |
| Steatosis (LFF) | 0.02 (0.01-0.07) | 0.01 (0.01-0.03) | 0.02 (0.01-0.04) | 0.087 |
| VAT (cm^3^) | 158 (99-187) | 151 (93-204) | 141 (86-197) | 0.980 |
| Abd SAT (cm^3^) | 348 (271-493) | 296 (177-431) | 255 (191-481) | 0.284 |
| HOMA-IR | 1.68 (1.1-2.73) | 2.52 (1.64-4.1) | 2.61 (1.13-5.78) | 0.111 |
| **Liver-related** |  |  |  |  |
| AST | 18 (15, 22) | 22 (17-33) | 29 (23-51) | <0.001 |
| ALT | 16 (14, 21) | 20 (13-27) | 23 (16-34) | 0.016 |
| Platelet count | 268 (235, 308) | 243 (202-285) | 204 (160-256) | 0.002 |
| FIB-4 | 0.82 (0.59, 0.97) | 1.03 (0.79-1.59) | 1.66 (1.17-3.02) | <0.001 |
| Presence of cirrhosis | 0 (0%) | 3 (6%) | 10 (22%) | 0.001 |
| **HIV-related factors**** |  |  |  |  |
| HIV RNA (copies/mL) | 45 (20, 93) | 80 (20-293) | 80 (34-2900) | 0.122 |
| HIV RNA undetectable | 12 (60%) | 17 (45%) | 18 (42%) | 0.39 |
| CD4 (/microL) | 640 (567, 781) | 554 (331-693) | 437 (212-638) | 0.04 |
| CD4 nadir | 555 (234, 929) | 241 (114-395) | 155 (104-288) | <0.001 |
| % currently on HAART | 19 (95%) | 31 (82%) | 32 (74%) | 0.15 |
| History of clinical AIDS | 6 (30%) | 16 (42%) | 25 (58%) | 0.09 |

Abbreviations: AA=African American, AIDS=acquired immunodeficiency syndrome, APRI=AST to platelet ratio index, AST=aspartate aminotransferase, ALT=alanine aminotransferase, FIB-4=fibrosis-4 score, HAART=highly active antiretroviral therapy, HCV=hepatitis C virus, HIV=human immunodeficiency virus, HOMA-IR=Homeostatic Model Assessment of Insulin Resistance, IDU=injection drug use, IQR=interquartile range, KTR=kynurenine:tryptophan ratio, LFF=liver fat fraction, RNA=ribonucleic acid, SAT=subcutaneous adipose tissue, U/L=units per liter, VAT=visceral adipose tissue

* Each KTR tertile contains one-third of the study population.  Specifically, tertile 1 represents the lowest third (KTR<0.0329), tertile 2 represents the middle third (KTR 0.0329-0.0457), and tertile 3 represents the highest third (KTR > 0.0457). P-values were calculated using T-test or Kruskal Wallis test for continuous and chi-squared test or Fisher’s exact test for categorical variables.

**Analysis restricted to HIV-positive persons only for all HIV-related factors except CD4 nadir
