# Supplementary material for: High Kynurenine:Tryptophan Ratio Is Associated With Liver Fibrosis in HIV-Monoinfected and HIV/Hepatitis C Virus–Coinfected Women
Source: Open Forum Infect Dis. 2019 Jun 11;6(7):ofz281. doi: 10.1093/ofid/ofz281 (PMC6612851; doi:10.1093/ofid/ofz281)
Supplement: ofz281_suppl_supplementary_table_2 [file ofz281_suppl_supplementary_table_2.docx]

Supplementary table 2. Factors associated with LSM in the total cohort in multivariable analysis

|  | **Adjusted model** | | **Adjusted model with KTR** | |
| --- | --- | --- | --- | --- |
| **Variables** | **% effect (95% CI)** | **p value** | **% effect (95% CI)** | **p value** |
| **Demographics** |  |  |  |  |
| Age (per 10 years) | 1% (-12%-15%) | 0.944 | -6% ( -18%-8%) | 0.362 |
| Race |  |  |  |  |
| African American (vs white) | 8% (-22%-49%) | 0.636 | 17% ( -14%-58%) | 0.324 |
| Hispanic (vs white) | 64% (8%-149%) | 0.02 | 63% (10%-142%) | 0.015 |
| Other (vs white) | 27% (-20%-101%) | 0.303 | 27% ( -17%-96%) | 0.273 |
| **Metabolic factors** |  |  |  |  |
| VAT | -5% (-18%-11%) | 0.523 | -5% (-18%-9%) | 0.459 |
| Steatosis (LFF) | 6% (-3%-15%) | 0.207 | 7% (-2%-16%) | 0.123 |
| **Lifestyle factors** |  |  |  |  |
| 0-7 drinks (vs none) | -13% (-30%-8%) | 0.219 | -11% (-27%-9%) | 0.272 |
| 7-12 drinks (vs none) | 7% (-37%-83%) | 0.803 | 12% (-33%-86%) | 0.673 |
| >12 drinks (vs none) | 1% (-35%-56%) | 0.978 | 8% (-29%-63%) | 0.730 |
| Current smoker | -12% (-32%-13%) | 0.305 | -13% (-31%-11%) | 0.262 |
| Current marijuana use | 18% (-6%-48%) | 0.164 | 13% (-9%-40%) | 0.272 |
| IDU ever | 6% (-23%-44%) | 0.728 | 4% (-22%-39%) | 0.787 |
| **Infection-related factors** |  |  |  |  |
| HIV monoinfection | 5% (-19%-37%) | 0.711 | -1% (-23%-27%) | 0.941 |
| HIV/HCV coinfection | 94% (40%-170%) | <0.001 | 47% (3%-110%) | 0.034 |
| KTR | N/A | N/A | 43% (15%-79%) | 0.002 |

Abbreviations: CI=confidence interval, HCV=hepatitis C virus, HIV=human immunodeficiency virus, IDU=injection drug use, KTR=kynurenine:tryptophan ratio, LFF=liver fat fraction, VAT=visceral adipose tissue
